# Handling and packaging of medical bags at acute disaster sites under high-temperature conditions

**DOI:** 10.1186/s13104-020-05014-4

**Published:** 2020-03-16

**Authors:** Wataru Ando, Yumika Imamura, Hideyuki Nagashima, Kouji Kondo, Kazunori Nakamura, Katsuya Otori

**Affiliations:** 1 Department of Clinical Pharmacy, Center for Pharmacy Practice and Sciences, School of Pharmacy, Kitasato University, 5-9-1 Shirokane, Minato-ku, Tokyo, 108-8641 Japan; 2 Department of Pharmacy, Kitasato University Medical Center, 6-100 Arai, Kitamoto, 364-8501 Saitama Japan; 3 Department of Neurosurgery, Kitasato University Medical Center, 6-100 Arai, Kitamoto, Saitama 364-8501 Japan; 4 Department of Surgery, Kitasato University Medical Center, 6-100 Arai, Kitamoto, Saitama 364-8501 Japan

**Keywords:** Disaster medical assistance team, Disaster medicine, Drug management

## Abstract

**Objective:**

After the large-scale earthquake in 2011, the disaster medical assistance team (DMAT) was made responsible for medical activities during the hyperacute phase of a disaster or accident in Japan. The medicines to be administered at the disaster sites, packaged in medical bags, may be affected by the temperatures there. This study aimed at establishing a method to handle drug bags in high-temperature situations by determining the temperature changes in medical bags subject to high temperatures and examining the effect of opening the bag and using heat-insulating material (HIM) and coolants.

**Results:**

Closed and semi-opened bags limited the temperature increase in the central part of the bag at both 35 and 40 °C to a greater extent than opened bags. When coolant and HIM were used in closed and semi-opened bags, the internal temperatures were significantly lower than in the opened state at 40 °C. In high-temperature disaster sites, medical bags should be maintained in a semi-opened or closed state using a HIM and coolant.

## Introduction

The medical teams dispatched to the site of a disaster or accident receive specialized training to work in the acute phase where they may be dealing with casualties with multiple injuries. In Japan, the system is based on the disaster medical assistance team (DMAT) [1]. After the large-scale earthquake in 2011 [2], the DMAT was responsible for medical activities during the hyperacute phase, and training of disaster medical teams is ongoing [3]. As these teams will need to administer medicines at the disaster sites, supplies are packaged in medical bags that can be carried to wherever they are needed. The stability of the medicines will differ, and some may be affected by high temperatures or low temperatures, which may reduce their effect [4]. However, drug management at the disaster site has not yet been fully evaluated. The disaster medical team knows empirically that the use of coolants and cold storage boxes is useful for temperature control of pharmaceuticals, but there is no evidence to show any definite measures.

This study aimed to determine the temperature change in medical bags subjected to high temperatures and also to examine the effect of opening the bag and using heat insulating material (HIM) and coolants. The purpose was to propose a method for handling drug bags in high temperature situations.

## Main text

### Materials and methods

#### Materials

The medical bags used were WJK-1C (Wako Shoji Co., Ltd., Kanagawa, Japan), which were 41 cm long, 49 cm wide, and 23 cm high, with fasteners on three sides. The bag volume was 44 L, and the weight was approximately 2.8 kg. The outer surface was polyvinyl chloride with an inner nylon lining; a 5 mm urethane sheet cushioned the top, sides, and bottom of the bags. Four small nylon bags that did not contain shock absorbent material were housed inside the bag. Dummy medicines were used in the same quantity, volume, weight, and shape as the 29 standard medicines defined by the DMAT Secretariat of the Ministry of Health, Labour, and Welfare of Japan (Additional file 1: Table S1). The total weight of the packed medical bag was approximately 12 kg, including ampules, soft bags, and packaging. A 10 mm expanded polystyrene foam board (Addtec, Tochigi, Japan) was used as the HIM; 500 g ice packs were used as the coolant (Ice Japan Co., Ltd., Hokkaido, Japan), which were frozen at − 20 °C before use, and four packs were used per bag. Heating was performed using a convection oven (Espec Corp., Osaka, Japan) as a thermostat box; the temperature was assessed using the MJ-UDL-22 temperature logger (Sato Shoji Inc., Kanagawa, Japan). The thermal images were captured using a thermal imaging camera (Type IR0004, PerfectPrime, Salford, United Kingdom).

### Methods

**Fig. 1:**
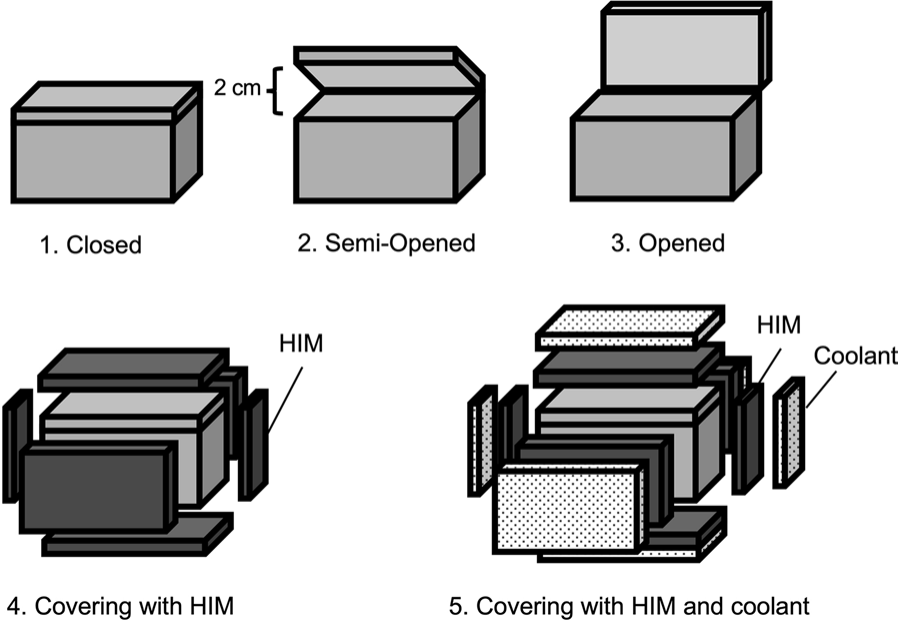
The three different medical bag states used in the experiment

The bag and dummy drugs were maintained at a constant room temperature of 24.5 ± 1.0 °C at least 24 h prior to the study. The temperature logger was placed in the center of the bag, and the dummy drugs and the temperature logger were packed in an identical manner for all experiments. As shown in Fig. 1, the bag was set in one of the following three ways: completely closed using the fasteners (closed); fasteners open on three sides, with the lid open partially without exposing the inside of the bag to the outside (semi-opened); or with the lid open and the inside of the bag fully exposed (opened). For each bag status, heat retention was evaluated without HIM and coolant, with HIM only, and with both HIM and coolant. The thermal images of the bags at 35 °C and 40 °C were compared using the thermal imaging camera and differences in the internal temperature of each bag was compared before and after 300 min of heating.

Initially, the temperature of the thermostat box was set to 35 °C or 40 °C and stabilized for 1 h before the experiment. After the bag was placed in the thermostat box, the temperature was measured at the start of the experiment and then after 15, 30, 45, and 60 min and every 30 min thereafter until 300 min had elapsed. Each experiment was performed three times. The goal was to maintain the temperature in the bag at 15–30 °C.

#### Statistical analysis

Student’s t-test was used to compare the two different temperature groups. For comparison of the three bag status groups, analysis of variance (ANOVA) was used. If the ANOVA was significant, a priori Fisher’s least significant difference (LSD) tests were used. A value of P < 0.05 was considered significant.

### Results

#### Comparison of the temperature in the medical bags in different states

**Fig. 2:**
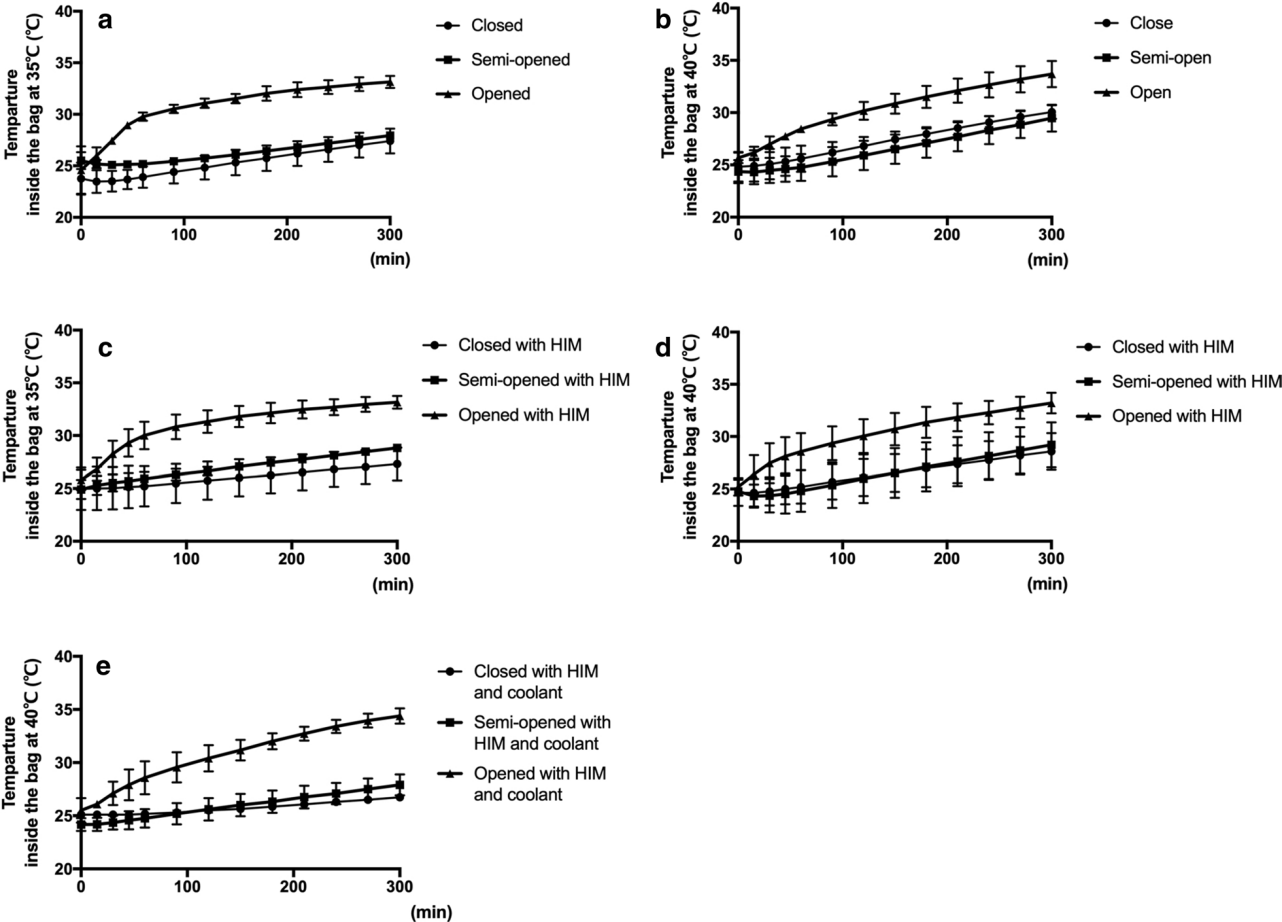
Comparison of temperature changes in medical bags in a closed, semi-opened, and opened state, with or without HIM and coolant. **a** 35 °C without HIM and coolant. **b** 40 °C without HIM and coolant. **c** 35 °C with HIM. **d** 40 °C with HIM. **e** 40 °C with HIM and coolant

After 300 min at 35 °C or 40 °C, the largest increase in the internal bag temperature was seen in the opened group (Fig. 2a, b). The temperature did not differ between the closed and semi-opened groups after exposure to 35 °C or 40 °C temperatures.

#### Use of heat-insulating materials and coolant

After 300 min, the temperature of the bag with HIM was lower than that of the bag without HIM in all bag statuses. At 35 °C, the bag with HIM was able to maintain a temperature of less than 30 °C (Fig. 2c). However, at 40 °C, the inside temperature exceeded 30 °C after 300 min (Fig. 2d). At 40 °C, the combination of HIM and the coolant maintained the temperature below 30 °C in the closed and semi-opened groups but not in the opened group (Fig. 2e).

#### Comparison of the internal temperature with the thermal imaging camera

**Fig. 3:**
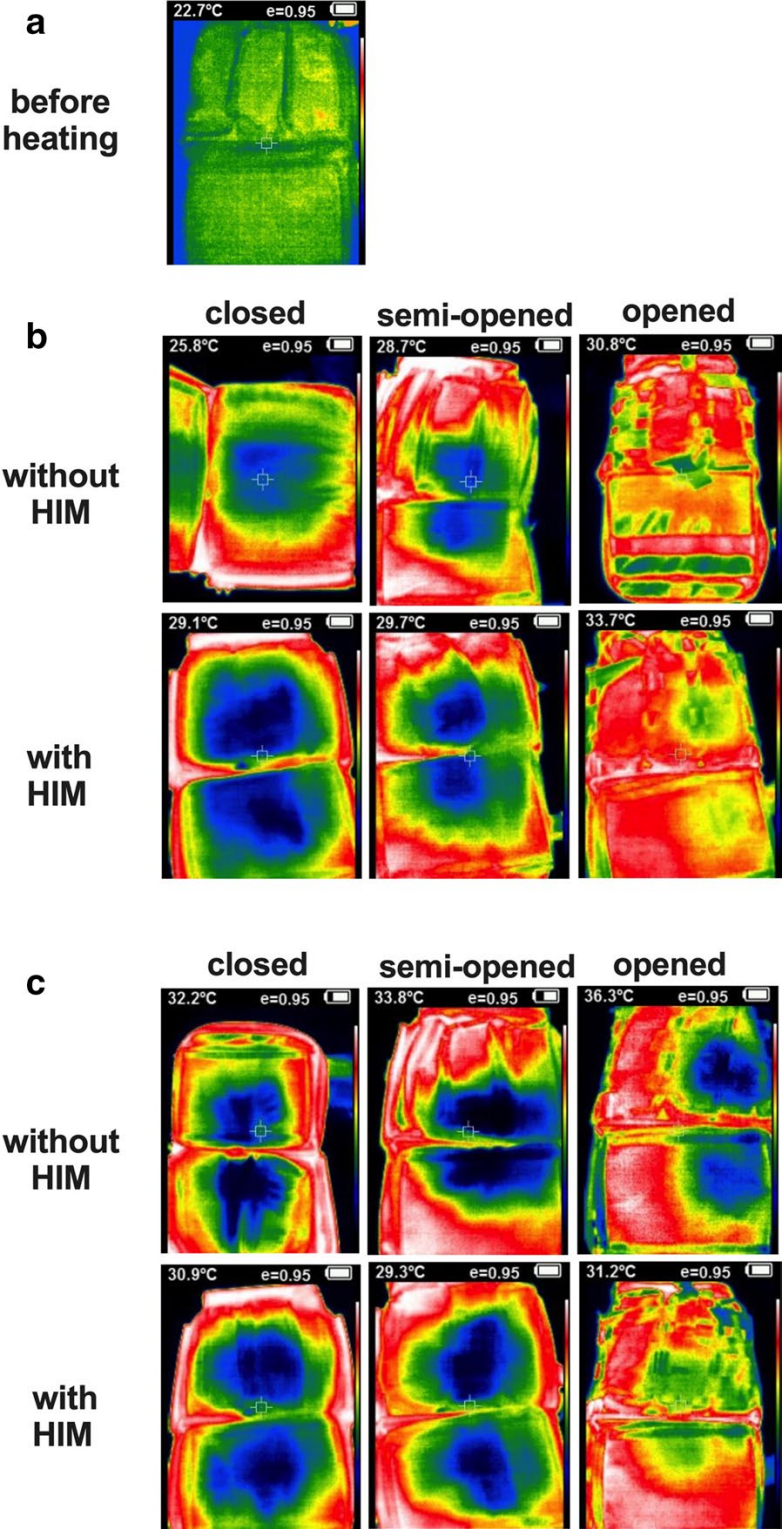
Thermal image of each bag after heating. The colors in the infrared image indicate the relative infrared energy and not the absolute temperature. Green indicates the center of the temperature, white and red indicate higher temperatures than the center and blue and black indicate lower temperatures. The temperature of each image indicates the temperature of the pixel at the center of the image. Before heating, the bag was uniformly green throughout. After heating, the temperature around the bag was relatively high, and conversely, the center temperature was low

Figures 3a, b illustrate the thermal images in each bag before and after heating them to 35 °C. After heating, the inside temperature of the closed and semi-opened bags was kept lower than the surface of the bag. The opened bags tended to have high internal temperatures, and this trend was also observed at 40 °C (Fig. 3c). These results indicate that there was a difference in temperature transfer time from the bag surface to the interior.

### Discussion

The results of this study show that an opened bag is more susceptible to the outside temperature than closed and semi-opened bags; also, closed and semi-opened bags are more likely to benefit from the use of HIM and coolant.

In this study, a 1 cm-thick expanded polystyrene plate was used as HIM, which showed more effective. Boards thinner than 1 cm were not used due to strength issues. However, because of the reduced internal volume, either larger bags may be required, or the number of medications may need to be reduced. Whereas the effectiveness of HIM was not sufficient when the bag was open to keep the temperature between 15 and 30 °C, it did provide protection from higher temperatures in the closed or semi-opened state. In addition to polystyrene foam, HIM includes an air cap, which consists of a resin sheet containing air particles. The air cap was not assessed in this study because the thickness of this layer changes when stacked. HIM is lightweight and inexpensive and can be readily adapted to the shape of the bag.

At the disaster site, the bag would need to be opened when in use, but the current study suggests that a semi-opened state would be preferable to maintain the temperature inside the bag. If the bag is not used for a long time at high temperatures, it should remain closed or semi-opened. As an alternative, drugs that are particularly temperature-sensitive should be placed in the center of the bag. In addition, if a medicine is extremely vulnerable to high temperatures, it may be necessary to manage it separately using a dedicated cooling box and a coolant.

The types of heat transfer are direct convection, conduction, and radiation [5]. Heat transfer through conduction and convection affects cold-chain insulated containers with phase change material [6]. In the current study, heat convection consists of the circulation of heated ambient air. The passage of hot air from outside the bag can be blocked, and convection can be suppressed by packing items evenly in the bag. Direct heat conduction can come from the ground, floor, and walls, as well as the heated environment. In the summer, paved areas of the ground are typically heated by sunlight, and the temperature can be higher than that of the environment. Therefore, medical bags should not be placed on the ground or flooring. The main source of heat radiation is sunlight, and it is therefore recommended that medical bags be placed in the shade. Particular care will be required for bags stored in vehicles when the air conditioning is not in use.

## Limitations

There are several limitations to the current study. The temperature in a disaster situation is not constant and will fluctuate. Also, the duration of time at 35 °C or 40 °C may exceed 5 h, for example, inside a tent. In addition, this study assessed temperature changes on a single day and did not evaluate temperature increases over 2 or more days. Other weather conditions, such as rain, were also not considered. The list of medicines also varies according to the type and scale of disasters, so the findings of this study may not be applicable to all disaster scenarios.

## Supplementary information

**Additional file 1: Table S1.** Based on 29 medicines by the DMAT Secretariat of the Ministry of Health, Labor, and Welfare of Japan.

## Data Availability

All data generated or analysed during this study are included in this published article and its supplementary information files.

## Abbreviations

HIM: Heat-insulating material
DMAT: Disaster medical assistance team
