# Supplementary material for: Handling and packaging of medical bags at acute disaster sites under high-temperature conditions
Source: BMC Res Notes. 2020 Mar 16;13:158. doi: 10.1186/s13104-020-05014-4 (PMC7077025; doi:10.1186/s13104-020-05014-4)
Supplement: Supplementary file 1 — Additional file 1: Table S1. Based on 29 medicines by the DMAT Secretariat of the Ministry of Health, Labor, and Welfare of Japan. [file 13104_2020_5014_MOESM1_ESM.docx]

Table S1. The based on 29 medicines by the DMAT Secretariat of the Ministry of Health, Labor and Welfare of Japan.

| Medicine | Volume (mL)* | | Dosage form | Provided  Number |
| --- | --- | --- | --- | --- |
| Normal saline solution | 500 |  | Plastic Bag | 3 |
| Ringer's solution | 500 |  | Plastic Bag | 5 |
| 20% D-mannitol injection solution | 300 |  | Plastic Bottle | 1 |
| 10% povidone iodine solution | 250 |  | Plastic Bottle | 1 |
| 7% sodium bicarbonate injection solution | 250 |  | Plastic Bag | 1 |
| 0.3% dopamine hydrochloride injection solution 600 mg | 200 |  | Plastic Bag | 1 |
| Normal saline solution | 100 |  | Plastic Bag | 5 |
| 0.5 mol magnesium sulfate injection solution | 20 |  | Ampoule | 5 |
| 20% glucose solution | 20 |  | Plastic ampoule | 4 |
| 5% glucose solution | 20 |  | Plastic ampoule | 5 |
| 7% sodium bicarbonate injection solution | 20 |  | Plastic ampoule | 5 |
| Distilled water for injection | 20 |  | Plastic ampoule | 10 |
| Ketamine hydrochloride intravenous injection 200 mg | 20 |  | Vial | 1 |
| Normal saline solution | 20 |  | Plastic ampoule | 10 |
| 1% lidocaine injection for topical hemp | 10 |  | Plastic ampoule | 10 |
| Nitroglycerin sublingual spray 0.3 mg | 8 |  | Spray | 1 |
| 2% lidocaine intravenous injection syringe | 5 |  | Prefiled syringe | 3 |
| Calcium polystyrene sulfonate 5 g | 5 |  | Powder | 12 |
| Procaterol Hydrochloride 10 μg Aerosol spray | 5 |  | Aerosol spray | 1 |
| Vecuronium bromide for intravenous injection solution 10 mg | 5 |  | Vial | 3 |
| Methylprednisolone sodium succinate 125 mg | 2 |  | Vial and ampoule | 5 |
| Midazolam Injection solution | 2 |  | Ampoule | 5 |
| Nicardipine hydrochloride injection solution 2 mg | 2 |  | Ampoule | 5 |
| Verapamil hydrochloride intravenous injection 5 mg | 2 |  | Ampoule | 3 |
| 0.05% atropine sulfate solution syringe | 1 |  | Prefiled syringe | 3 |
| 0.1% epinephrine injection syringe | 1 |  | Prefiled syringe | 5 |
| Buprenorphine hydrochloride injection solution 0.2 mg or Pentazocine Injection solution 15 mg | 1 |  | Ampoule | 10 |
| Diazepam Injection solution 5 mg | 1 |  | Ampoule | 5 |
| Diazepam suppository 10 mg | 1 | g | Suppository | 5 |

*Exclude the weight of the outer packaging
